# SPIKES clinical protocol for delivering bad news: an experience report

**DOI:** 10.1590/0034-7167-2024-0638

**Published:** 2025-12-08

**Authors:** Michel Siqueira da Silva, Micheline Veras de Moura, Andrea Maria Magalhães de Andrade, Karoline Queiroz Martins Almeida de Araújo

**Affiliations:** I Universidade Federal do Rio Grande do Norte. Natal, Rio Grande do Norte, Brazil; II Universidade de Coimbra. Coimbra, Portugal; III Centro Universitário Unidade de Ensino Superior Dom Bosco. São Luís, Maranhão, Brazil

**Keywords:** Communication, Clinical Protocols, News, Palliative Care, Nursing., Comunicación, Protocolos Clínicos, Noticias, Cuidados Paliativos, Enfermería.

## Abstract

**Objectives:**

to apply the SPIKES protocol in a family meeting with a patient undergoing palliative care for metastatic cholangiocarcinoma.

**Methods:**

an experience report from a teaching hospital. The SPIKES protocol was used to communicate bad news in six steps.

**Results:**

communication mediated by the SPIKES protocol strengthened the bond between health team, patient, and their family members. Furthermore, there was a reduction in family members’ emotional distress and greater acceptance of clinical decisions, which directly reflected the trust placed in the care team.

**Final Considerations:**

the SPIKES protocol proved to be an essential tool for promoting palliative care. More than strengthening bonds, it contributed to shared and humanized decisions, favoring the dignity of the dying process. Moreover, it reinforces the need for training healthcare professionals, emphasizing the ethical and empathetic role of the care team. Information confidentiality was guaranteed, ensuring the anonymity of those involved.

## RESUMEN

**Objetivos:**

aplicar el protocolo SPIKES en una reunión familiar con un paciente en cuidados paliativos por colangiocarcinoma metastásico.

**Métodos:**

informe de experiencia en un hospital universitario. Se utilizó el protocolo SPIKES para comunicar malas noticias en seis etapas.

**Resultados:**

la comunicación mediada por el protocolo SPIKES fortaleció el vínculo entre el equipo de salud, el paciente y sus familiares. Además, se observó una reducción del malestar emocional en los familiares y una mayor aceptación de las decisiones clínicas, lo que refleja directamente la confianza depositada en el equipo de atención.

**Consideraciones Finales:**

el uso del protocolo SPIKES ha demostrado ser una herramienta esencial para promover los cuidados paliativos. Además de fortalecer vínculos, ha contribuido a la toma de decisiones compartidas y humanizadas, favoreciendo la dignidad del proceso de morir. Además, refuerza la necesidad de capacitar a los profesionales de la salud, destacando el rol ético y empático del equipo de atención. Se garantizó la confidencialidad de la información, asegurando el anonimato de los involucrados.

## INTRODUCTION

The natural cycle of human life encompasses events such as birth, growth, reproduction and, finally, death. However, for many, death remains shrouded in diverse perceptions, opinions and interpretations, making it difficult to access and understand^(1)^. The way each individual assimilates the reality of death and deals with this moment is influenced by their personal and professional experiences, which determines differences in the way patients cope with and respond to the process of finitude^(2)^.

Palliative care is a fundamental approach for patients facing life-threatening illnesses, such as advanced cholangiocarcinoma. According to the World Health Organization, palliative care aims to improve the quality of life of patients and their families through early relief of distress and rigorous management of physical, emotional, social and spiritual symptoms^(3)^. In Brazil, this type of care has advanced in recent decades, but it still faces challenges related to the fragility of training for healthcare professionals as well as awareness and involvement of family members regarding the specificities and singularities of the topic^(4)^.

In the context of oncology, palliative care takes on particular importance due to the physical and emotional impact that the disease causes. Metastatic cholangiocarcinoma, in particular, is characterized by an aggressive clinical course and, often, with few curative therapeutic options available^(5)^. In light of this, the focus of care shifts to symptom control and comprehensive support for patients and their families, providing dignity and comfort throughout the illness process. This transition, related to end-of-life care, requires clear, empathetic and respectful communication between healthcare team and patients’ families^(5)^.

In this scenario, it is important to highlight that delivering more news is characterized as any communication that, directly or indirectly, causes a negative change in patients’ lives, according to their perception, generating potentially traumatic implications and sensations. This is a frequent practice in healthcare professionals’ routine that requires skill and sensitivity to minimize the emotional impacts involved^(6)^.

Therefore, delivering bad news in particular is one of the most challenging aspects faced by healthcare professionals, especially in oncology. From this perspective, the SPIKES protocol^(7)^ is widely used to guide communication in complex and sensitive situations, such as in palliative care settings. The protocol proposes a six-step approach: Setting (preparing the environment); Perception (patient and family perception); Invitation (invitation to discuss); Knowledge (sharing information); Emotions (emotional response); and Strategy and Summary (strategy and care plan). Studies show that applying this protocol can reduce family members’ anxiety and improve their understanding of patients’ clinical condition^(7,8)^.

The importance of effective and structured communication in palliative care is widely recognized as a pillar to ensure humanized and patient-centered care^(4,9)^. Throughout the report, we highlighted the challenges and strategies used by the team, focusing on the role of nurses as a mediator and facilitator of communication.

## OBJECTIVES

To describe, through an experience report, the application of the SPIKES clinical protocol during a family meeting of a patient with metastatic cholangiocarcinoma, in palliative care, conducted in an Oncohematology Unit of a teaching hospital in northeastern Brazil, in addition to highlighting the importance of structured and humanized communication in the care of terminally ill patients and their families.

## METHODS

### Ethical aspects

According to the Brazilian National Health Council, through Resolution 466/2012, which governs the guidelines and regulatory standards for research involving human beings in Brazil, some types of studies do not require approval from a Research Ethics Committee (REC), especially when the research does not involve a risk to participants’ integrity or is not considered scientific research, but rather an experience report.

Thus, this study deals with the application of the SPIKES protocol in a family meeting of a patient in palliative care, and is therefore a report of care experience based on already consolidated practices. Therefore, as the study does not involve the manipulation of sensitive data or new experimental interventions that may directly impact patients, the protocol does not require prior assessment by the REC, in accordance with article 2, item IV, of Resolution 466/2012.

### Study design

This is a descriptive and qualitative experience report that aims to describe the importance of the SPIKES protocol in delivering bad news in palliative care. The qualitative approach was chosen because it allows for an in-depth understanding of the subjective aspects involved in the communication process between healthcare professionals, patients and family members. For organization and transparency in the study description, the COnsolidated criteria for REporting Qualitative research checklist guidelines were followed.

### Methodological procedures

#### Study setting

This clinical practice narrative took place in a teaching hospital in northeastern Brazil, with a 50-year-old patient diagnosed with metastatic cholangiocarcinoma in palliative care. The multidisciplinary team, composed of a palliative care physician, nurse, social worker and psychologist, were responsible for conducting the family meeting.

#### Data source

The data source was direct, non-participant observation of the application of the SPIKES protocol during a family meeting with a patient in palliative care. The choice of setting was intentional, as it represents a real situation of delivering bad news, enabling the analysis of the interaction between health team, patient and family members.

Observation focused on communicational dynamics, emotional reactions and reception strategies adopted by professionals. The records were kept in a structured field diary, respecting the principles of non-interference and ensuring the ethical rigor of the qualitative approach.

#### Data collection and organization

Data collection was performed through participant observation during the meeting, in which communication and family reactions were recorded. Observations were recorded in a structured field diary immediately after the meeting, respecting the principles of non-interference in the communication process. The recording focused on communication dynamics, emotional reactions, reception strategies and interventions by the multidisciplinary team, seeking to ensure report fidelity. In addition, the quality of the protocol in communication and family support was documented. Data were organized based on the SPIKES protocol steps (Setting, Perception, Invitation, Knowledge, Emotions, and Strategy and Summary).

#### Work steps

- Team preparation: before the meeting, the multidisciplinary team met to review patients’ medical history, define the objectives of the meeting, and plan strategies to deal with the family’s emotional reactions. This initial preparation period was important to align information and define the responsibilities of each team member during the conversation;
- Scheduling and choosing the location: the meeting was scheduled according to the family’s availability and took place in a private location, outside the ward, with the aim of providing comfort and safety to all involved, as recommended by the SPIKES protocol^(7)^;
- Application of the SPIKES protocol: during the meeting, the six steps of the SPIKES protocol were carefully followed, with a focus on active listening and emotional support for family members. The nurse played a key role in mediating the dialogue, clarifying doubts and offering emotional support to those present. The protocol, widely recognized in clinical practice for its structured and empathetic approach, guided the multidisciplinary team actions, composed of a palliative care physician, nurse, social worker and psychologist, to ensure clear and respectful communication with patients and their family members;
- Post-meeting analysis: after the meeting, family members’ reactions, level of understanding of patients’ clinical situation, and effectiveness of the emotional support provided were analyzed. Family members’ perceptions of the clinical condition were explored to ensure a shared understanding of the situation, while the multidisciplinary team worked in an integrated manner to provide clear information, considering the compromised emotional limits, and to build an individualized and patient-centered care plan.

#### Data analysis

Data analysis was carried out based on qualitative categories, namely:

- Emotional support: how much the team was able to offer emotional support to family members during the meeting;
- Clarity of communication: the degree to which family members understand patients’ clinical situation and the next steps for care;
- Decision support: how the team facilitated the construction of a patient-centered care plan.

Subcategories for analysis include: family members’ perceptions of the clinical picture, which were explored to ensure a shared understanding of the situation; and the multidisciplinary team’s integrated work to provide clear information, considering the compromise of emotional limits, and to build an individualized and patient-centered care plan.

The meeting began with the Setting step, in which a private and welcoming environment was prepared to ensure participant comfort and emotional safety. A palliative care physician began the conversation by introducing herself and inviting everyone present to do the same, in order to create an atmosphere of respect and empathy. This was followed by a moment of active listening, in which family members were encouraged to share their perceptions of patients’ clinical condition. This step, corresponding to the SPIKES protocol’s Perception step, revealed different levels of understanding and expectation, ranging from family members who were hopeful for a cure to others who were already showing signs of understanding the severity of the condition.

During the Invitation step, a physician asked family members about their willingness to listen to information provided about patients’ situation. This moment was renewed delicately, respecting each person’s emotional limits. With the consent of those present, the team moved on to the Knowledge step, in which the evolution of patients’ clinical condition and the impossibility of curative treatment were explained clearly and empathetically. The nurse played a fundamental role in translating complex medical terms into accessible and clear language, clarifying the family members’ doubts that arose during this step, providing greater understanding and reassurance about the uncertainty of the scenario described regarding patients’ condition.

The Emotions step was marked by several conversations among family members, including crying, a specific emotional reaction that suggests emotional genuineness in the context of delivering bad news. Moreover, issues about patients’ future and feelings of anger and frustration were addressed. At this point, the team demonstrated empathy, respecting each demonstration and offering emotional support.

From this perspective, a nurse, a specialist in palliative care, dedicated himself to welcoming the most intense relationships, validating the feelings expressed and reinforcing the commitment to providing relief to patients’ distress^(7)^. This welcome was essential to strengthen the bond between the team and family members, reducing the emotional impact of the information transmitted.

In the final step, Strategy and Summary, the multidisciplinary team developed an individualized care plan for patients focusing on symptom control, psychological support, and ongoing care. The team presented the plan collaboratively, allowing family members to participate in decisions and express their concerns. The active involvement of those present contributed to the flexibility of the proposed strategies and reinforced the importance of patientand family-centered care. Furthermore, the importance of continued monitoring by the multidisciplinary team and ensuring support for any needs that arise, such as the suspension of disproportionate therapeutic measures that prolong patients’ distress but do not provide significant clinical benefits, was discussed. The application of the SPIKES protocol provided a respectful and humanized dialogue, allowing family members to understand patients’ clinical context and feel supported in the decision-making process. The conflicts initially presented were mitigated through active listening, transparency, and emotional support provided by the team, highlighting the central role of nurses as a mediator and facilitator of compassionate care.

## DISCUSSION

This clinical practice record highlights the importance of applying the SPIKES protocol in delivering bad news in palliative care settings, especially in advanced oncology scenarios. Recent studies from 2024 corroborate the relevance of this protocol, indicating that its use promotes clearer, more empathetic and structured communication, which is significant for building a bond of trust with family members and for humanizing care^(4,8)^. It is understood that the emotional impact caused by the news that a curative treatment is unfeasible can be mitigated when a healthcare team, guided by protocols such as SPIKES, acts in an integrated manner, offering emotional support and transparent information.

In the oncological context, transition to palliative care often involves multiple challenges, both for patients and their family members, who may have unrealistic expectations and intense emotions^(10)^. The highlighted report, as well as the application of the SPIKES protocol steps, especially Perception and Emotions, was fundamental to dealing with the social and emotional conflicts present. By allowing family members to express their perceptions and reactions, the team was able to promote horizontal and welcoming communication, a point emphasized in several experience reports published recently^(9)^.

Another relevant aspect addressed by recent studies is the main role of nurses as a mediator in communication in palliative care. As evidenced in the report, the presence of a nurse as a facilitator, mediator between medical terms and the family, and responsible for welcoming those involved, with regard to emotional support, is crucial to the success of the family meeting. Nurses trained in palliative care have considerable capacity to humanize communication, ensuring that family members understand the information in an accessible way and feel welcomed in critical moments^(6)^. This reflects the importance of continuing training for nursing professionals so that they can develop their skills and competencies to deal with the emotional and social demands of patients and their families, especially when delivering bad news.

Furthermore, the use of the SPIKES protocol reinforces the need for care centered not only on patients, but also on their family members, an approach that has been widely discussed in recent publications^(11)^. Involving family members in the development of a care plan is an effective strategy for strengthening therapeutic decisions and promoting a collaborative and humanized approach. Open communication and inclusion of family members in decision-making are key elements for successful palliative care and for reducing the emotional impact^(8)^.

In summary, this report highlights the importance of structured and humanized communication in the context of palliative care, highlighting how the SPIKES protocol promotes empathetic and effective communication among the health team. In addition, it highlights the importance of training and qualification of professionals for the application of protocols such as SPIKES. This report can contribute to effective and humanized communication, thus avoiding conflicts, promoting acceptance and ensuring care focused on the needs and desires of patients and their family members, reflecting the commitment to dignity and comfort in times of vulnerability.

Studies reinforce that delivering bad news requires technical and empathetic skills, which can be achieved through specific training^(4)^.

## FINAL CONSIDERATIONS

The family meeting highlighted the central role of nurses as a mediator of communication and facilitator of acceptance of family members’ emotions. Active listening, translation of technical terms into accessible language and an empathetic approach were fundamental strategies to strengthen the bond between the health team and the family. This responsibility reinforces the need for continuing training, especially for working in palliative care contexts, ensuring a humanized practice centered on patients and their families.

The experience of delivering bad news based on the SPIKES protocol reinforces the importance of applying standardized and structured instruments in palliative care contexts. The use of this protocol proved to be essential to ensure clear, respectful and empathetic communication, allowing family members to adequately understand the transition from health to illness in the context of palliative care.

It is concluded that the adoption of evidence-based communication approaches not only improves the approach to care, but also strengthens the therapeutic relationship and shared decision-making. Thus, it highlights the need for integration in healthcare professionals’ training and practice.

### Study limitations

This study has limitations that are inherent to its design, as it is a unique experience, in an exclusive context, in which there is no control over external variables, which may have influenced the reported outcomes.

### Contributions to nursing, health or public policy

This study offers a significant contribution to nursing, highlighting the importance of humanized and effective communication in assisting patients in palliative care and their families. In terms of public policies, this work suggests the inclusion of communication approaches in training and in guidelines for care practices, thus contributing to palliative care in health systems.

## Data Availability

The research data are available only upon request.
