# Supplementary material for: SPIKES clinical protocol for delivering bad news: an experience report
Source: Rev Bras Enferm. 2025 Dec 8;78(6):e20240638. doi: 10.1590/0034-7167-2024-0638 (PMC12707526; doi:10.1590/0034-7167-2024-0638)
Supplement: Translation

# Translation: Protocolo clínico SPIKES na comunicação de más notícias: relato de experiência

**DOI:** 10.1590/0034-7167-2024-0638pt

**Authors:** Michel Siqueira da Silva, Micheline Veras de Moura, Andrea Maria Magalhães de Andrade, Karoline Queiroz Martins Almeida de Araújo

**Affiliations:** I Universidade Federal do Rio Grande do Norte. Natal, Rio Grande do Norte, Brasil; II Universidade de Coimbra. Coimbra, Portugal; III Centro Universitário Unidade de Ensino Superior Dom Bosco. São Luís, Maranhão, Brasil

**Keywords:** Comunicação, Protocolo Clínico, Notícias, Cuidados Paliativos, Enfermagem.

## Abstract

**Objetivos:**

aplicar o protocolo SPIKES em reunião familiar em paciente em cuidados paliativos por colangiocarcinoma metastático.

**Métodos:**

relato de experiência em hospital de ensino. Utilizou-se o protocolo SPIKES por meio da comunicação de más notícias em seis etapas.

**Resultados:**

a comunicação mediada pelo protocolo SPIKES fortaleceu o vínculo entre a equipe de saúde, paciente e seus familiares. Além disso, observou-se redução do sofrimento emocional dos familiares e maior aceitação das decisões clínicas, refletindo diretamente na confiança depositada na equipe assistencial.

**Considerações Finais:**

o uso do protocolo SPIKES demonstrou ser uma ferramenta essencial para a promoção de cuidados paliativos. Mais do que fortalecer vínculos, contribuiu para decisões compartilhadas e humanizadas, favorecendo a dignidade do processo de morrer. Além disso, reforça-se a necessidade de capacitação de profissionais de saúde, ressaltando-se o papel ético e empático da equipe assistencial. Foram garantidos o sigilo e a confidencialidade das informações, assegurando o anonimato dos envolvidos.

## INTRODUÇÃO

O ciclo natural da vida humana abrange eventos como o nascimento, o crescimento, a reprodução e, por fim, a morte. No entanto, para muitos, a morte permanece envolta de diversas percepções, opiniões e interpretações, dificultando a sua acessibilidade e compreensão^(1)^. A forma como cada indivíduo assimila a realidade da morte e lida com esse momento é influenciada por suas experiências pessoais e profissionais, o que determina diferenças no enfrentamento e na resposta dos pacientes ao processo de finitude^(2)^.

Os cuidados paliativos são uma abordagem fundamental para pacientes que enfrentam doenças que ameaçam a continuidade da vida, como o colangiocarcinoma em estágio avançado. Para a Organização Mundial da Saúde, os cuidados paliativos visam melhorar a qualidade de vida dos pacientes e de suas famílias por meio do alívio precoce sofrimento e manejo rigoroso de sintomas físicos, emocionais, sociais e espirituais^(3)^. No Brasil, essa modalidade de cuidado avançou nas últimas décadas, mas ainda enfrenta desafios relacionados à fragilidade da formação de profissionais de saúde, bem como à conscientização e ao envolvimento dos familiares quanto às especificidades e singularidades sobre a temática^(4)^.

No contexto oncológico, os cuidados paliativos ganham uma importância particular devido ao impacto físico e emocional que a doença causa. O colangiocarcinoma metastático, em especial, é caracterizado por um curso clínico agressivo e, muitas vezes, com poucas opções terapêuticas curativas disponíveis^(5)^. Diante disso, o foco da assistência se desloca para o controle dos sintomas e suporte integral ao paciente e aos seus familiares, proporcionando dignidade e conforto durante todo o processo de adoecimento. Essa transição, relacionada aos cuidados de fim de vida, requer uma comunicação clara, empática e respeitosa entre a equipe de saúde e a família do paciente^(5)^.

Nesse cenário, é importante destacar que a transmissão de mais notícias é caracterizada como qualquer comunicação que, de forma direta ou indireta, provoque uma mudança negativa na vida do paciente, conforme a sua percepção, gerando implicações e sensações potencialmente traumáticas. Trata-se de uma prática frequente na rotina dos profissionais da saúde que requer habilidade e sensibilidade para minimizar os impactos emocionais envolvidos^(6)^.

Portanto, em particular, a comunicação de más notícias é um dos aspectos mais desafiadores enfrentados pelos profissionais de saúde, sobretudo na oncologia. Sob essa perspectiva, o protocolo SPIKES^(7)^ é amplamente utilizado para orientar a comunicação em situações complexas e sensíveis, como em contextos de cuidados paliativos. O protocolo propõe uma abordagem em seis etapas: *Setting* (preparação do ambiente); *Perception* (percepção do paciente e dos familiares); *Invitation* (convite para a discussão); *Knowledge* (compartilhamento de informações); *Emotions* (resposta emocional); e *Strategy and Summary* (estratégia e plano de cuidados). Estudos mostram que a aplicação desse protocolo pode reduzir a ansiedade dos familiares e melhorar a sua compreensão sobre o quadro clínico do paciente^(7,8)^.

A importância da comunicação eficaz e estruturada nos cuidados paliativos é extremamente reconhecida como um pilar para garantir um cuidado humanizado e centrado no paciente^(4,9)^. Ao longo do relato, destacamos os desafios e as estratégias utilizadas pela equipe, com foco no papel do enfermeiro como mediador e facilitador da comunicação.

## OBJETIVOS

Descrever, por meio de relato de experiência, a aplicação do protocolo clínico SPIKES durante uma reunião familiar de um paciente com colangiocarcinoma metastático, em cuidados paliativos, conduzido em uma Unidade de Oncohematologia de um hospital de ensino no Nordeste brasileiro, além de evidenciar a importância de uma comunicação estruturada e humanizada na assistência a pacientes em fase terminal e a seus familiares.

## MÉTODOS

### Aspectos éticos

De acordo com o Conselho Nacional de Saúde, por meio da Resolução n^o^ 466/2012, que rege as diretrizes e normas regulamentadoras de pesquisas envolvendo seres humanos no Brasil, alguns tipos de estudos não exigem a aprovação de um Comitê de Ética em Pesquisa (CEP), especialmente quando a pesquisa não envolve risco à integridade dos participantes ou não é considerada uma pesquisa científica, mas sim um relato de experiência.

Dessa forma, este estudo trata da aplicação do protocolo SPIKES em uma reunião familiar de um paciente em cuidados paliativos, tratando-se, portanto, de um relato de vivência assistencial baseado em práticas já consolidadas. Portanto, como o estudo não envolve manipulação de dados sensíveis ou novas intervenções experimentais que possam impactar diretamente o paciente, o protocolo não necessita de uma avaliação prévia do CEP, conforme o artigo 2º, inciso IV, da Resolução n^o^ 466/2012.

### Tipo de estudo

Trata-se de relato de experiência, de natureza descritiva e qualitativa, que visa descrever a importância do protocolo SPIKES na comunicação de más notícias em cuidados paliativos. A abordagem qualitativa foi escolhida por permitir compreender, em profundidade, os aspectos subjetivos envolvidos no processo comunicacional entre profissionais de saúde, pacientes e familiares. Para organização e transparência na descrição do estudo, foram seguidas as diretrizes do *checklist COnsolidated criteria for REporting Qualitative research*, recomendado para relatos qualitativos.

### Procedimentos metodológicos

#### Cenário do estudo

Esta narrativa de prática clínica ocorreu em um hospital de ensino no nordeste do Brasil, com um paciente de 50 anos de idade, diagnosticado com colangiocarcionoma metastático, em cuidados paliativos. A equipe multidisciplinar, composta por médico paliativista, enfermeiro, assistente social e psicólogo, foi responsável pela condução da reunião familiar.

#### Fonte de dados

A fonte de dados foi a observação direta, do tipo não participante, da aplicação do protocolo SPIKES durante uma reunião familiar com paciente em cuidados paliativos. A escolha do cenário foi intencional, por representar uma situação real de comunicação de más notícias, possibilitando a análise da interação entre equipe de saúde, paciente e familiares.

A observação teve como foco os aspectos comunicacionais, reações emocionais e estratégias de acolhimento adotadas pelos profissionais. O registro foi realizado em diário de campo estruturado, respeitando os princípios da não interferência e assegurando o rigor ético da abordagem qualitativa.

#### Coleta e organização de dados

A coleta de dados foi realizada por meio de observação do participante na reunião, na qual a comunicação e as reações dos familiares foram registradas. As observações foram anotadas em diário de campo estruturado, imediatamente após o encontro, respeitando os princípios da não interferência no processo comunicacional. O registro focou na dinâmica comunicacional, reações emocionais, estratégias de acolhimento e intervenções da equipe multiprofissional, buscando garantir a fidelidade do relato. Além disso, a qualidade do protocolo na comunicação e no apoio familiar foi documentada. Os dados foram organizados com base nas etapas do protocolo SPIKES (*Setting, Perception, Invitation, Knowledge, Emotions* e *Strategy and Summary*).

#### Etapas do trabalho

- Preparação da equipe: antes da reunião, a equipe multidisciplinar se reuniu para revisar o histórico clínico do paciente, definir os objetivos do encontro e planejar as estratégias para lidar com as reações emocionais dos familiares. Esse momento inicial de preparação foi importante para alinhar as informações e definir as responsabilidades de cada membro da equipe durante a condução da conversa;
- Agendamento e escolha do local: a reunião foi agendada de acordo com a disponibilidade da família e ocorreu em um local reservado, fora da enfermaria, com o objetivo de proporcionar conforto e segurança a todos os envolvidos, conforme preconizado pelo protocolo SPIKES^(7)^;
- Aplicação do protocolo SPIKES: durante a reunião, as seis etapas do protocolo SPIKES foram seguidas cuidadosamente, com foco na escuta ativa e no acolhimento emocional dos familiares. O enfermeiro desempenhou um papel fundamental na mediação do diálogo, esclarecendo dúvidas e oferecendo suporte emocional aos presentes. O protocolo, amplamente reconhecido na prática clínica por sua abordagem estruturada e empática, orientou as ações da equipe multidisciplinar, composta por médico paliativista, enfermeiro, assistente social e psicólogo, para garantir uma comunicação clara e respeitosa com o paciente e os seus familiares;
- Análise pós-reunião: após a reunião, foram analisadas as reações dos familiares, o nível de compreensão sobre a situação clínica do paciente e a eficácia do suporte emocional oferecido. As percepções dos familiares sobre o quadro clínico foram exploradas para garantir uma compreensão compartilhada da situação, enquanto a equipe multidisciplinar trabalhava de forma integrada para oferecer informações claras, considerando o comprometimento dos limites emocionais, e construir um plano de cuidados individualizado e centrado no paciente.

#### Análise dos dados

A análise dos dados foi realizada com base em categorias qualitativas, sendo elas:

- Acolhimento emocional: o quanto a equipe foi capaz de oferecer suporte emocional aos familiares durante a reunião;
- Clareza na comunicação: o grau de compreensão dos familiares sobre a situação clínica do paciente e os próximos passos para o cuidado;
- Apoio à decisão: como a equipe facilitou a construção de um plano de cuidados centrado no paciente.

As subcategorias para para análises incluem: as percepções dos familiares sobre o quadro clínico que foram exploradas para garantir uma compreensão compartilhada da situação; e o trabalho integrado da equipe multidisciplinar para oferecer informações claras, considerando o comprometimento dos limites emocionais, e construir um plano de cuidados individualizado e centrado no paciente.

A reunião teve início com a etapa *Setting*, em que foi preparado um ambiente privado e acolhedor para garantir o conforto e a segurança emocional dos participantes. Uma médica paliativista iniciou a conversa, apresentando-se e convidando todos os presentes a fazerem o mesmo, de modo a criar um clima de respeito e empatia. Em seguida, foi realizado um momento de escuta ativa, no qual os familiares foram encorajados a compartilhar suas percepções sobre o quadro clínico do paciente. Essa etapa, correspondente à *Perception* do protocolo SPIKES, revelou diferentes níveis de compreensão e expectativa, variando de familiares com esperanças de cura a outros que já demonstravam sinais de entendimento da gravidade do quadro.

Durante a etapa *Invitation*, a médica questionou os familiares sobre sua disposição para ouvir informações fornecidas sobre a situação do paciente. Esse momento foi renovado com delicadeza, respeitando os limites emocionais de cada um. Com o consentimento dos presentes, a equipe passou para a etapa *Knowledge*, na qual foram expostas, de forma clara e empática, a evolução do quadro clínico do paciente e a impossibilidade de um tratamento curativo. O enfermeiro desempenhou um papel fundamental de traduzir termos médicos complexos para uma linguagem acessível e clara, ao esclarecer as dúvidas dos familiares surgidas nessa etapa, proporcionando maior compreensão e segurança à incerteza do cenário descrito sobre a condição do doente.

A etapa *Emotions* foi marcada por diversas conversas entre os familiares, incluindo choro, uma reação emocional específica que sugere genuinidade emocional no contexto de comunicação de más notícias. Além disso, foram abordados questionamentos sobre o futuro do paciente e os sentimentos de raiva e frustração dos envolvidos. Neste momento, a equipe demonstrou empatia, respeitando cada demonstração e oferecendo suporte emocional.

Sob essa perspectiva, o enfermeiro, especialista em cuidados paliativos, dedicou-se a acolher as relações mais intensas, validando os sentimentos expressos e reforçando o compromisso de proporcionar alívio ao sofrimento do paciente^(7)^. Esse acolhimento foi fundamental para fortalecer o vínculo entre a equipe e os familiares, reduzindo o impacto emocional das informações transmitidas.

Na etapa final, *Strategy and Summary*, foi elaborado um plano de cuidados individualizado pela equipe multidisciplinar para o paciente com foco no controle de sintomas, suporte psicológico e assistência contínua. A equipe apresentou o plano de forma colaborativa, permitindo que os familiares participassem das decisões e expressassem suas preocupações. O envolvimento ativo dos presentes contribuiu para a flexibilidade das estratégias propostas e reforçou a importância de um cuidado centrado no paciente e na família. Além disso, foram discutidas a importância da continuidade do acompanhamento pela equipe multidisciplinar e a garantia de suporte em todas as necessidades que surgirem, como a suspensão de medidas terapêuticas desproporcionais que prologam o sofrimento do paciente, mas sem benefícios clínicos significativos. A aplicação do protocolo SPIKES proporcionou um diálogo respeitoso e humanizado, permitindo que os familiares compreendessem o contexto clínico do paciente e se sentissem apoiados no processo de tomada de decisão. Os conflitos apresentados inicialmente foram mitigados por meio da escuta ativa, da transparência e do suporte emocional oferecido pela equipe, destacando o papel central do enfermeiro como mediador e facilitador de um cuidado compassivo.

## DISCUSSÃO

Este registro de prática clínica destaca a importância da aplicação do protocolo SPIKES na comunicação de más notícias em contextos de cuidados paliativos, especialmente em cenários oncológicos avançados. Estudos recentes de 2024 corroboram a relevância desse protocolo, apontando que sua utilização promove uma comunicação mais clara, empática e estruturada, o que é significativo para a construção de um vínculo de confiança com os familiares e para a humanização do cuidado^(4,8)^. Compreende-se que o impacto emocional causado pela notícia da inviabilidade de um tratamento curativo pode ser atenuado quando uma equipe de saúde, orientada por protocolos como o SPIKES, atua de maneira integrada, oferecendo suporte emocional e informações transparentes.

No contexto oncológico, a transição para os cuidados paliativos frequentemente envolve múltiplos desafios, tanto para o paciente quanto para seus familiares, que podem apresentar expectativas irreais e emoções intensas^(10)^. O relato destacado, bem como a aplicação das etapas do protocolo SPIKES, em especial a *Perception* e *Emotions*, foi fundamental para lidar com os conflitos sociais e emocionais presentes. Ao permitir que os familiares expressassem suas percepções e reações, a equipe conseguiu promover uma comunicação horizontal e acolhedora, um ponto enfatizado em diversos relatos de experiência publicados recentemente^(9)^.

Outro aspecto relevante abordado por estudos recentes é a atribuição principal do enfermeiro como mediador na comunicação em cuidados paliativos. Conforme evidenciado no relato, a presença do enfermeiro como facilitador, mediador entre os termos médicos e a família, e responsável pelo acolhimento dos envolvidos no que diz respeito ao suporte emocional é determinante para o sucesso da reunião familiar. Os enfermeiros com formação em cuidados paliativos têm capacidade considerável de humanizar a comunicação, garantindo que os familiares compreendam as informações de maneira acessível e sintam-se acolhidos em momentos críticos^(6)^. Isso reflete a importância da capacitação contínua dos profissionais enfermeiros, de forma que possam desenvolver suas habilidades e competências para lidar com as demandas emocionais e sociais dos pacientes e de suas famílias, em especial na comunicação de más notícias.

Além disso, o uso do protocolo SPIKES reforça a necessidade de um cuidado centrado não apenas no paciente, mas também em seus familiares, uma abordagem que vem sendo amplamente discutida em publicações recentes^(11)^. O envolvimento dos familiares na construção do plano de cuidados é uma estratégia eficaz para fortalecer as decisões terapêuticas e promover uma abordagem colaborativa e humanizada. A comunicação aberta e a inclusão dos familiares nas decisões são elementos-chave para o sucesso dos cuidados paliativos e para a redução do impacto emocional^(8)^.

Em síntese, este relato evidencia a importância de uma comunicação estruturada e humanizada no contexto de cuidados paliativos, destacando como o protocolo SPIKES promove uma comunicação empática e eficaz entre a equipe de saúde. Além disso, destaca-se a importância da formação e capacitação de profissionais para a aplicação de protocolos como o SPIKES. Este relato pode contribuir com uma comunicação eficaz e humanizada, para, assim, evitar conflitos, promover acolhimento e garantir um cuidado centrado nas necessidades e nos anseios do paciente e dos seus familiares, refletindo o compromisso com a dignidade e o conforto em momentos de vulnerabilidade.

Estudos reforçam que a comunicação de más notícias requer habilidades técnicas e empáticas, que podem ser realizadas por meio de treinamentos específicos^(4)^.

## CONSIDERAÇÕES FINAIS

A condução da reunião familiar evidenciou o papel central do enfermeiro como mediador da comunicação e facilitador do acolhimento das emoções dos familiares. A escuta ativa, a tradução de termos técnicos em uma linguagem acessível e a abordagem empática foram estratégias fundamentais para fortalecer o vínculo entre a equipe de saúde e a família. Essa responsabilidade reforça a necessidade de capacitação contínua, sobretudo para atuar em contextos de cuidados paliativos, assegurando uma prática humanizada e centrada no paciente e na família.

A experiência da comunicação de más notícias baseada no protocolo SPIKES reforça a importância da aplicação de instrumentos padronizados e estruturados em contextos de cuidados paliativos. O uso desse protocolo mostrou ser fundamental para garantir uma comunicação clara, respeitosa e empática, permitindo que os familiares compreendessem de maneira adequada a transição do estado saúde-doença em contexto de cuidados paliativos.

Conclui-se que a adoção de abordagens comunicacionais baseadas em evidências não apenas melhora a abordagem dos cuidados, mas também fortalece a relação terapêutica e a tomada de decisão compartilhada. Assim, evidencia a necessidade de integração na formação e prática dos profissionais de saúde.

### Limitações do estudo

Este estudo tem limitações que são inerentes ao delineamento, por se tratar de uma experiência única, em um contexto exclusivo, em que não há controle de variáveis externas, que podem ter influenciado os desfechos relatados.

### Contribuições para as áreas da enfermagem, saúde ou políticas públicas

Este estudo oferece uma contribuição significativa para enfermagem, destacando a importância de uma comunicação humanizada e eficaz na assitência ao paciente em cuidados paliativos e aos seus familiares. Em termos de políticas públicas, este trabalho sugere a inclusão de abordagens comunicacionais na formação e nas diretrizes de práticas assistenciais, contribuindo, assim, para os cuidados paliativos nos sistemas de saúde.

## Data Availability

Os dados de pesquisa só estão disponíveis mediante solicitação.
